# Effects of prophylactic indomethacin on morbidity and mortality in infants <25 weeks’ gestation: a protocol driven intention to treat analysis

**DOI:** 10.1038/s41372-022-01547-7

**Published:** 2022-10-30

**Authors:** Ronald I. Clyman, Nancy K. Hills

**Affiliations:** 1 Department of Pediatrics, University of California San Francisco, San Francisco, CA USA; 2 Cardiovascular Research Institute, University of California San Francisco, San Francisco, CA USA; 3 Department of Epidemiology and Biostatistics, University of California San Francisco, San Francisco, CA USA; 4 Department of Neurology, University of California San Francisco, San Francisco, CA USA

**Keywords:** Respiratory tract diseases, Peripheral vascular disease

## Abstract

**Objective:**

To determine if prophylactic indomethacin (PINDO) decreases death or bronchopulmonary dysplasia-grades 2 and 3 (death/BPD) in newborns <25 weeks.

**Study design:**

Intention-to-treat, cohort-controlled study of 106 infants admitted during three alternating epochs of PINDO or Expectant patent ductus arteriosus (PDA) management.

**Results:**

At 7–8 days 85% of Expectant Management epoch infants had a moderate/large PDA (median exposure was 23 days). Among PINDO epoch infants only 24% still had a PDA at 7–8 days. There were no significant differences in the incidence of death/BPD or of secondary outcomes (BPD, death, necrotizing enterocolitis/spontaneous perforations, or intraventricular hemorrhage (grades 3 or 4)) in either unadjusted or adjusted comparisons between infants born in a PINDO epoch and those born in the Expectant Management epoch.

**Conclusion:**

Despite being at high risk for PDA-related morbidities, PINDO did not appear to alter the rates of our primary and secondary outcomes in infants <25 weeks.

## Introduction

In premature infants, bronchopulmonary dysplasia (BPD) is a significant problem with long-term effects on growth and neurodevelopment [1]. Infants with grades 2 or 3 BPD (as defined by Jensen et al. [2]) are reported to have a 47% chance of having late death or serious childhood respiratory morbidity compared with infants with no BPD (10%) or mild, grade 1 BPD (19%) [2].

Whether early prophylactic treatment of patent ductus arteriosus (PDA) with nonsteroidal anti-inflammatory drugs (NSAIDs, e.g., indomethacin and ibuprofen) can decrease the incidence of BPD is still a matter of debate. In preclinical studies, early prophylactic NSAID treatment prevented some of the aberrant alveolar development that occurs when preterm baboons are invasively ventilated for 2 weeks after birth [1, 3]. On the other hand, randomized controlled clinical trials (RCTs) have not found a reduction in the incidence of BPD when prophylactic or early pre-symptomatic PDA treatment has been compared with more conservative Expectant Management approaches [4, 5]. However, when the RCTs were initially designed there was little information about the natural history of the PDA, its relationship to BPD, and who might be at risk for PDA-related morbidities. Subsequent studies have shown that the association between PDA and BPD depends on the magnitude and duration of the PDA shunt and on an interaction between the PDA and the duration of invasive ventilation [6–11]. Although the presence of a PDA increases the risk of BPD, this only appears to occur when the shunt is moderate/large and persists for longer than 7–14 days. In addition, the increased risk of BPD (especially the more severe forms of BPD) only seems to occur in infants who require invasive ventilation for at least 10 days in addition to being exposed to the moderate/large PDA shunt. The incidence of BPD seems to be unaffected by prolonged PDA exposures in infants who require <10 days of invasive ventilation [7, 9]. Unfortunately, RCTs performed before the year 2000 paid little attention to the magnitude and duration of the PDA shunt or the infants’ need for respiratory support. More recent RCTs [12–16] have ensured that infants enrolled in the trials have moderate/large PDA at the time of enrollment. However, even in these RCTs, the exposure to invasive ventilation has been relatively brief (median duration <7 days), well below the 10 days exposure threshold which appears to alter the relationship between PDA and BPD [7, 9]. As such, the study populations of most RCTs have included infants for whom the presence of a persistent PDA might not be an important risk factor and where closing the PDA may have little or no effect on the incidence of BPD.

Although the clinical RCTs have not shown a benefit for early PDA treatment, several studies using large retrospective databases have reported improved neonatal mortality and morbidity in nurseries that use early PDA screening and/or treatment [17, 18]. In addition, a meta-analysis involving more than 11,000 very preterm infants found that prophylactic indomethacin (PINDO) was associated with a significant, albeit small, reduction in neonatal death [19]. Several recent more granular analyses of observational databases have concluded that PINDO may act in a gestational age-dependent manner with beneficial effects in the sickest, most immature infants (i.e., those below 750 grams birthweight and below 25 weeks’ gestation) [20–24] and harmful effects in healthier, more mature preterm infants [22, 23]. Unfortunately, as has been pointed out previously [25], the infrequent and idiosyncratic use of PINDO in the observational studies just referenced makes their PINDO-treated infants a highly selected subpopulation that likely suffers from clinician selection bias.

Few studies have examined the effects of PINDO in a population comprised exclusively of infants <25 weeks’ gestation (those at highest risk for PDA-related morbidities due to their frequent prolonged exposures to a PDA and prolonged need for invasive ventilation). We, therefore, performed a cohort-controlled study in infants <25 weeks’ gestation using data from an ongoing quality improvement project that has been examining neonatal outcomes associated with different protocol-driven PDA treatment approaches. We hypothesized that infants born in an epoch when PINDO was started within 24 h of birth unless contraindicated (PINDO epoch) would have a lower incidence of the composite outcome of death or BPD than infants born in an epoch where Expectant Management (no treatment for first 7 postnatal days to allow for spontaneous PDA closure) was the protocol approach. Since the PINDO and Expectant Management approaches were both assigned by protocol/epoch, we anticipated that our analysis would not be confounded by indication as has been the case in prior observational studies.

## Methods

### Patient population and PINDO and expectant management protocols

Our primary goal was to determine if routine PINDO treatment of infants born before 25 weeks’ gestation altered the incidence of the composite outcome BPD (grades 2 or 3) or death before 36 weeks.

We compared two groups of infants: those admitted during an epoch where all were eligible for PINDO treatment and those admitted during an epoch where all received Expectant Management and none received PINDO. Infants were included in the study population if they delivered before 25 weeks’ gestation, were admitted to the intensive care nursery within 24 h of birth and survived beyond 24 h. The study was approved by the Institutional Review Board of the University of California San Francisco and was performed in accordance with the Declaration of Helsinki.

Infants born before 25 weeks’ gestation were not frequent admissions to our nursery; it took 17 years to enroll the study’s 106 infants (from 2005 and 2022). Since it was likely that some clinical practices or demographic variables might change during the time span of the consecutive study treatment epochs, we attempted to minimize differences between the PINDO and Expectant Management groups by placing the Expectant Management epoch in the middle of the study period and bracketing the Expectant Management epoch with two PINDO epochs – one at the beginning and one at the end of the study.

During epoch 1 (PINDO epoch (A), January 2005 to April 2011), all infants were started on a course of PINDO within 24 h of birth (provided there were no contraindications such as ongoing coagulopathy, concurrent use of hydrocortisone for hypotension, or oliguria (urine < 1 ml/kg/hour). Five potential PINDO doses were given at 24 h intervals (a 0.2 mg/kg loading dose followed by two to four 0.1 mg/kg maintenance doses). Maintenance doses 3 and 4 were given only if there was evidence (even minimal) of ductus patency on an echocardiogram performed after the second maintenance dose. All infants had an echocardiogram performed on postnatal days 7 or 8.

During epoch 2 (Expectant Management epoch, May 2011 to June 2017) PINDO was no longer used and no infant was treated with indomethacin during the first 7 days to allow for spontaneous PDA closure [26]. During epoch 2, all infants had an echocardiogram on postnatal days 7 or 8.

During epoch 3 (PINDO epoch (B), July 2017 to June 2022), we returned to the same PINDO approach outlined in PINDO epoch (A). All infants had an echocardiogram on postnatal days 7 or 8.

During all three study epochs the same echocardiographic criteria were used to define the presence of a moderate/large PDA. The echocardiographic criteria that were used at both 7 to 8 days and throughout the hospitalization included: an internal PDA diameter ≥1.5 mm (or PDA:left pulmonary artery diameter ratio ≥0.5) in addition to one or more of the following criteria: (a) left atrium-to-aortic root ratio ≥1.6, (b) ductus flow velocity ≤2.5 m/sec or mean pressure gradient across the ductus ≤8 mm Hg, (c) left pulmonary artery diastolic flow velocity >0.2 m/sec, and/or (d) reversed diastolic flow in the descending aorta [6, 9, 27]. During the PINDO and Expectant Management epochs, moderate/large PDAs that persisted beyond 7 days could be treated pharmacologically with indomethacin or acetaminophen. The decision to treat a moderate/large PDA after the first week was left to the infant’s clinical team. PDAs that did not meet the definition of moderate/large PDA were considered to be “constricted” (small or closed) and were never treated.

During the PINDO and Expectant Management epochs, infants with a persistent moderate/large PDA after the first week were followed with frequent (every 7–14 days) echocardiograms to determine when ductus constriction occurred. Infants with a “constricted” PDA were followed less frequently (every 2-3 weeks) until ductus closure or hospital discharge.

The duration of exposure to a moderate/large PDA was calculated and expressed in days as previously described [6, 9, 27]. The day of birth was considered day 0. Infants with small or closed ductus at postnatal day 7 were assumed to have had a constricted ductus during the first 7 days. Infants with moderate/large PDAs at postnatal day 7 were assumed to have been exposed to a moderate/large PDA for the entire 7 days. The time of ductus constriction was assumed to have occurred at the halfway point between the last exam with a moderate/large PDA and the first exam with a constricted ductus. When reopening of the PDA occurred after documented ductus constriction, the additional exposure to the reopened moderate/large PDA shunt was calculated as the number of days from the echocardiogram demonstrating the reopened moderate/large shunt to the time of ductus constriction (i.e., the halfway point between the last exam with a moderate/large PDA and the first exam with a constricted ductus). The duration of exposure to the reopened PDA was added to the initial moderate/large PDA shunt exposure.

### Risk factors and outcomes

A single neonatologist (RIC) prospectively evaluated and recorded all perinatal/neonatal risk factors and outcome measures during the hospitalization (Tables 1 and 2). Gestational age was determined by the date of last menstrual period and early ultrasounds (before 24 weeks gestation). Birthweight-for-gestational age z-scores were obtained using the growth curves from Fenton et al. [28]. “Small for gestational age” was defined as *z* < −1.29.

**Table 1:** Demographic risk factors of infants who were born in each of the three epochs (either Prophylactic Indomethacin (PINDO) epoch or expectant management).

Risk factors	Epoch 1 PINDO (*n* = 42)	Epoch 2 Expectant management (*n* = 38)	Epoch 3 PINDO (*n* = 26)
Prenatal variables			
Multiple Gestation - %	17	37	23
Preeclampsia - %	2	24	19
Maternal Diabetes - %	7	3	8
Chorioamnionitis - %	43	18	19
Antenatal Betamethasone - %: None <24 hours or >9 days prior to delivery ≥24 hours and ≤9 days prior to delivery	33 21 45	32 3 66	8 8 85
Antenatal Indomethacin within 10 days of birth - %	7	21	35
Caesarian section - %	40	71	54
Delayed Cord Clamping - %	0	42	73
Outborn - %	38	29	8
Neonatal variables			
Gestation – weeks (m ± sd)	24.3 ± 0.4	24.2 ± 0.2	24.4 ± 0.3
Gestation <24^0/7^ weeks - %	12	13	8
Birthweight – grams (m ± sd)	652 ± 121	632 ± 116	637 ± 116
Small for Gestational Age - %	14	18	19
White - %	38	37	38
Male - %	52	61	54
5 min Apgar ≤5 - %	31	54	54
PINDO received within 1st 24 h - %	90	0	92
Intubated in the delivery room - %	93	89	62
Intubated during 1st 24 h - %	93	100	73
Intubated for >24 h during first 3 days - %	83	92	54
Respiratory Severity Score (RSS) at 24 h^a^ - median (IQR)	1.8 (1.5, 2.5)	2.4 (2.0, 3.4)	2.2 (1.8, 2.8)
Days intubated during first 24 days - median (IQR)	13 (3, 19)	16 (4, 22)	9 (3, 14)
Dopamine ≥10 μgm/kg/min for >24 h during 1st 96 h - %	27	42	15
Fluid gain during 1st 72 h^b^ - ml/kg/day	70 ± 39	65 ± 31	58 ± 29
Bacteremia (culture positive) - %	33	39	42
PDA moderate/large at 7 days - %	18	85	30
PDA moderate/large at 14 days - %	15	75	30
Days exposed to moderate/large PDA^c^ - median (IQR)	0 (0, 8)	23 (8, 32)	0 (0, 16)
Outcomes			
Death before 36 weeks or BPD (grades 2 or 3) - %	52	55	42
Death during hospitalization - %	45	39	31
BPD (grades 2 or 3)^d^ - %	13	26	17
sIVH (grades 3 or 4)^e^ - %	32	35	19
NEC or SIP during hospitalization^f^ - %	21	29	33
Pulmonary Hemorrhage^g^ - %	15	10	4

^a^Respiratory Severity Score (RSS) at 24 h: Mean airway pressure x FiO2 at 24 h.

^b^Fluid gain during 1st 72 h: daily fluid intake minus urine output.

^c^Days exposed to moderate/large PDA: see Methods: Infants with a constricted ductus at postnatal day 7 were assumed to have had 0 days of moderate/large PDA exposure during the first 7 days.

^d^BPD (grades 2 or 3), bronchopulmonary dysplasia (grades 2 or 3). 64 infants could be evaluated for the outcome; 42 infants could not be fully evaluated because they died before the 36 weeks’ evaluation.

^e^sIVH (grades 3 or 4), 101 infants could be evaluated for intracranial hemorrhages (grades 3 or 4); 5 infants could not be fully evaluated because they died before 72 h.

^f^NEC or SIP during hospitalization, 74 infants could be evaluated for the outcome; 32 infants could not be fully evaluated because they died before 14 days.

^g^Pulmonary hemorrhage, 90 infants could be evaluated for the outcome; 16 infants could not be fully evaluated because they died before 5 days.

**Table 2:** Demographic risk factors of infants who were born in either a Prophylactic Indomethacin (PINDO) epoch or an Expectant Management epoch.

Risk factors	Epoch 2 expectant management (*n* = 38)	Epochs 1 & 3 PINDO (*n* = 68)
Prenatal variables		
Multiple Gestation - %	37	19^±^
Preeclampsia - %	24	9*
Maternal Diabetes - %	3	7
Chorioamnionitis - %	18	34
Antenatal Betamethasone - %: None <24 hours or >9 days prior to delivery ≥24 hours and ≤9 days prior to delivery	32 3 66	24^±^ 17 59
Antenatal Indomethacin within 10 days of birth - %	21	18
Caesarian section - %	71	46*
Delayed Cord Clamping - %	42	28
Outborn - %	29	26
Neonatal variables		
Gestation – weeks (m ± sd)	24.2 ± 0.2	24.3 ± 0.3
Birthweight – grams (m ± sd)	632 ± 116	647 ± 119
Small for gestational age - %	18	16
White - %	37	38
Male - %	61	53
5 min Apgar ≤5 - %	54	40
PINDO received within 1st 24 h - %	0	91****
Intubated in the delivery room - %	89	81
Intubated during 1st 24 h - %	100	85*
Intubated for >24 h during first 3 days - %	92	72*
Respiratory Severity Score (RSS) at 24 h^a^ - median (IQR)	2.4 (2.0, 3.4)	2.0 (1.6, 2.7)*
Days intubated during first 24 days - median (IQR)	16 (4, 22)	11 (3, 17)
Dopamine ≥10 μgm/kg/min for >24 h during 1st 96 h - %	42	22*
Fluid gain during 1st 72 h^b^ - ml/kg/day	65 ± 31	65 ± 35
Bacteremia (culture positive) - %	39	37
PDA moderate/large at 7 days - %	85	24****
PDA moderate/large at 14 days - %	75	21****
Days exposed to moderate/large PDA^c^ - median (IQR)	23 (8, 32)	0 (0, 13) ****
Outcomes		
Death or BPD (grades 2 or 3) - %	55	49
Death during hospitalization - %	39	40
BPD (grades 2 or 3)^d^ - %	26	15
sIVH (grades 3 or 4)^e^ - %	35	27
NEC or SIP during hospitalization^f^ - %	29	27
Pulmonary Hemorrhage^g^ - %	10	10

±*p* < 0.10; *p < 0.05; **p < 0.01; ***p < 0.005; ****p < 0.001

^a^Respiratory Severity Score (RSS) at 24 h: Mean airway pressure x FiO2 at 24 h.

^b^Fluid gain during 1st 72 h: daily fluid intake minus urine output.

^c^Days exposed to moderate/large PDA: see Methods: Infants with a constricted ductus at postnatal day 7 were assumed to have had 0 days of moderate/large PDA exposure during the first 7 days.

^d^BPD (grades 2 or 3), bronchopulmonary dysplasia (grades 2 or 3). 64 infants could be evaluated for the outcome; 42 infants could not be fully evaluated because they died before the 36 weeks’ evaluation.

^e^sIVH (grades 3 or 4), 101 infants could be evaluated for intracranial hemorrhages (grades 3 or 4); 5 infants could not be fully evaluated because they died before 72 h.

^f^NEC or SIP during hospitalization, 74 infants could be evaluated for the outcome; 32 infants could not be fully evaluated because they died before 14 days.

^g^Pulmonary hemorrhage, 90 infants could be evaluated for the outcome; 16 infants could not be fully evaluated because they died before 5 days.

#### Bronchopulmonary dysplasia

Our primary outcome was the composite outcome BPD (grades 2 or 3) or death before 36 weeks. All infants (except those requiring continuous positive nasal airway pressure (CPAP) with ≥30% oxygen or mechanical ventilation) underwent a modified room air challenge test between 36^0/7^ and 36^6/7^ weeks [29]. Those who failed the test (or who required CPAP with ≥30% oxygen or mechanical ventilation) were classified as “BPD” and were further classified as Grades 2 and 3 BPD (using the criteria of Jensen et al. [2]) if they required either nasal cannula flow rates >2 L/min, noninvasive positive airway pressure, or invasive mechanical ventilation [2].

#### Serious Intraventricular Hemorrhage (sIVH)

All infants were examined with serial bedside cranial ultrasounds initiated within the first week of life. Critically ill infants had their first examination on day 1 or 2. These were repeated weekly for the first 4 weeks until stable. Imaging was repeated prior to discharge or, more frequently, if there were any abnormal findings. Serious intraventricular hemorrhage, sIVH, was defined as grades 3 or 4 IVH (using the four-level grading system) [30]. Since the full extent of an IVH is often not evident until after day 4 [31, 32], we excluded infants who died without evidence of sIVH prior to day 4 from our IVH analyses.

#### Pulmonary hemorrhage

Serious pulmonary hemorrhages were defined by the presence of frank blood in the tracheal secretions, a sudden need for increased respiratory support, and chest X-ray changes. We excluded from our pulmonary hemorrhage analyses infants who died without evidence of pulmonary hemorrhage prior to day 5, since it was not until day 5 that at least 80% of the serious pulmonary hemorrhages became evident.

#### Necrotizing enterocolitis (NEC), perforated NEC (NEC-perf)), and spontaneous intestinal perforations (SIP)

NEC was defined as Bell’s classification II or greater (this included NEC that was treated medically or surgically). Intestinal perforations (either SIP or NEC-perf) were diagnosed by either the presence of a pneumoperitoneum on abdominal X-ray or by the surgeon at the time of laparotomy. Infants who died prior to 14 days without evidence of NEC or perforation (before a diagnosis could be made) were excluded from the analyses.

### Statistical analysis

Stata software (Release 17.0; StataCorp LP, College Station, Texas) was used for all statistical analyses. Chi-squared, Mann–Whitney *U* Test and Student’s *t*-tests were used to compare groups for categorical and parametric variables, respectively. We used multivariable logistic regression to build statistical models that could adjust for the possible confounding effects of the demographic risk factors on the relationship between our variable of interest (infants who were eligible for PINDO - i.e., those born in either of the PINDO epochs (epochs 1 and 3)) and the outcomes examined.

PINDO and Expectant Management approaches were assigned by epoch and therefore not confounded by indication. We used an intention-to-treat approach and included all infants in our analyses. PINDO was delayed or not given to nine percent of the infants born during the PINDO epochs (epochs 1 and 3) because of either ongoing coagulopathy, concurrent use of hydrocortisone for hypotension, or oliguria (Tables 1 and 2). Our results were considered exploratory and were not adjusted for multiple comparisons.

Since the observational period of our study spanned an interval of 17 years, we included a “birth year” variable in our models to adjust for unmeasured practice changes that may have occurred over time. To determine whether infants eligible for PINDO had a decrease in our primary outcome (BPD grades 2 or 3 or death) we first created a basic model that included our variable of interest (“PINDO epoch”) adjusted for year of birth. We performed a logistic regression to determine the odds ratio and 95% confidence interval (OR, 95% CI) of our primary outcome for the variable “*PINDO epoch”*. Next, we added each of the demographic variables listed in Table 2 to the basic model, one at a time, and reran the logistic regression to determine how much the OR for the variable “PINDO epoch” was altered by the addition of the new variable to the basic model. If the addition of the new variable altered the OR for the association between “PINDO epoch” and the outcome by more than 10% we considered it to be a variable “of potential interest” that should be added to the *final adjusted model* (i.e., if the OR for “PINDO epoch” was 0.9 in the basic model, a new variable would be considered “of potential interest” if the OR for “PINDO epoch” became <0.81 or >0.99 when the new variable was added to the model). We repeated this step for each of the demographic variables. We constructed the final adjusted model for “PINDO epoch” by adjusting the basic model for all demographic variables of interest. This final multivariable model was analyzed with logistic regression using generalized estimating equations techniques to account for clustering within multiple (twin/triplet) births (Table 3).

**Table 3:** Odds ratios (OR) and 95% confidence intervals (95% CI) for the primary and secondary outcomes.

Outcomes	Epoch 2 expectant management	Epochs 1 & 3 PINDO
	OR (95% CI)	OR (95% CI)
Primary outcome		
Death or BPD (grades 2 or 3)	1	1.16 (0.42, 3.18)
Secondary outcomes		
Death during hospitalization	1	2.36 (0.63, 8.78)
BPD (grades 2 or 3)	1	0.64 (0.10, 4.06)
sIVH (grades 3 or 4)	1	0.94 (0.30, 2.92)
NEC or SIP during hospitalization	1	2.05 (0.46, 9.19)

The OR (95% CI) for the variable of interest (PINDO epoch) in the Final Adjusted models is presented in the table. The other “demographic variables of interest” included in the Final Adjusted Models are presented below:

Death or BPD (grades 2 or 3): The final model was adjusted for: Year of birth, Respiratory Severity score at 24 h, Intubated for >24 h during first 3 days, and Dopamine ≥10 μgm/kg/min during 1st 96 h.

Death during the hospitalization: The final model was adjusted for: Year of birth, Antenatal betamethasone, Caesarian section delivery, Respiratory Severity score at 24 h, Intubated for >24 h during first 3 days, and Dopamine ≥10 μgm/kg/min during 1st 96 h.

BPD (grades 2 or 3): The final model was adjusted for: Year of birth, Caesarian section delivery, Apgar score at 5 min ≤5, Respiratory Severity score at 24 h, Number of Days intubated during first 24 days, and Dopamine ≥10 μgm/kg/min during 1st 96 h.

sIVH (grades 3 or 4): The final model was adjusted for: Year of birth, Antenatal betamethasone, Outborn birth, Respiratory Severity score at 24 h, Intubated for >24 h during first 3 days, and Dopamine ≥10 μgm/kg/min during 1st 96 h.

NEC or SIP during hospitalization: Year of birth, Preeclampsia, Caesarian section delivery, Apgar score at 5 min ≤5, Respiratory Severity score at 24 h, Intubated for >24 h during first 3 days, and Dopamine ≥10 μgm/kg/min during 1st 96 h.

We used the same stepwise approach described above to examine the effects of “PINDO epoch” on other secondary outcomes: death, BPD (grades 2 or 3), sIVH, and NEC/SIP.

## Results

One hundred eleven infants <25 weeks’ gestation were admitted during the study period. Our study population was composed of the 106 who survived beyond 24 h (Table 1).

Although the incidences of most demographic variables listed in Table 1 were not related to the infants’ year of birth during the study, a few variables did appear to be significantly associated with birth year: preeclampsia (OR (95% CI): 1.12 (1.03, 1.22)), chorioamnionitis (0.88 (0.80, 0.97)), and outborn birth (0.88 (0.81, 0.95))). In addition, several clinical practices underwent planned changes during the study period and their incidences were related to the year the infant was born; these included increased use of indomethacin for tocolysis (OR (95%CI): 1.19 (1.01, 1.40)), increased use of rescue betamethasone treatment for mothers who were ≥10 days beyond their first dose of betamethasone (1.16 (1.06, 1.26)), increased use of delayed umbilical cord clamping (1.62 (1.36, 1.94)), and increased preference given to non-invasive respiratory support and avoidance of tracheal intubation and ventilation at delivery (0.84 (0.73, 0.97)). There were no changes in our protocols for initiation and duration of caffeine administration or of feeding advances (data not shown). Probiotics and vitamin A were not used during the study period.

Ninety-one percent of the infants born during the PINDO epochs received prophylactic indomethacin treatment during the first 24 h. Only 24% of the infants born during the PINDO epochs had a moderate/large PDA shunt at the end of the first week (Table 2). In contrast, 85% of the Expectant Management epoch infants still had a moderate/large PDA shunt after the first week. Forty-five percent of the Expectant Management infants received indomethacin or acetaminophen as rescue PDA treatment after 7 days. The median duration of moderate/large PDA shunt exposure for all infants in the Expectant Management epoch was 23 days (Table 2).

There were no significant differences in the incidences of our primary outcome or any of the secondary outcomes in unadjusted comparisons between infants born during the PINDO epochs and those born during the Expectant Management epoch (Table 2). Despite positioning the PINDO epochs before and after the Expectant Management epoch (to minimize time-related differences between the treatment groups), some of the potentially confounding demographic variables still differed significantly between the two treatment approaches (Table 2). We created multivariable models to adjust for possible confounding effects on the relationships between “PINDO epoch” and the various outcomes. In our Final Adjusted models, we found no significant relationship between “PINDO epoch” and the incidence of BPD (grades 2 or 3) or death before 36 weeks (Table 3). Nor was there an association between “PINDO epoch” and death during the hospitalization, BPD (grades 2 or 3), sIVH, or episodes of SIP or NEC when compared with Expectant Management (Table 3).

## Discussion

Our study was designed to examine the effects of prophylactic indomethacin in infants <25 weeks’ gestation at high risk for PDA-related morbidities. The median duration of invasive ventilation in both treatment groups during the first 3.5 weeks was greater than 10 days. The median duration of PDA exposure in the Expectant Management group was 23 days; in contrast, only 24% of “PINDO epoch” infants still had a moderate/large PDA shunt after the first week (Table 2). Despite the high risk for PDA-related morbidities in our study population we did not find an association between PINDO and our primary outcome BPD (grades 2 or 3) or death before 36 weeks in either the unadjusted analysis or in our adjusted models; neither did we find an association between PINDO and any of the secondary outcomes (Table 3).

There are several limitations to our study. We used data from a single center. Since the incidence of moderate/large PDA and neonatal morbidities differ by center, our results may not be generalizable to centers where the rates differ from ours. Our study was not a randomized controlled trial. As an observational study, it cannot distinguish between causation and association and should be used primarily for hypothesis generation. Our study also spanned a 17 years’ interval. Even though our study was bookended by the PINDO epochs and our analyses were adjusted for the year of entry into the study and for potential confounding variables between the groups, there may have been unmeasured changes in practice that could have affected the morbidity rates during the study period. In addition, the relatively small size of our study may have made it difficult to detect significant differences for some of our study outcomes. Because only a limited number of infants were available for our study, we cannot exclude the possibility that our finding of no differences was due to insufficient power. However, our study size was necessarily limited by the small number of infants available for inclusion.

On the other hand, there are important strengths to our study. Few other studies have examined the effects of PINDO in a population comprised exclusively of infants <25 weeks’ gestation – those most at risk for potential PDA-related morbidities. Our protocol driven approach meant that the use of PINDO was only minimally confounded by indication or selection bias, which has been the major limitation of prior observational retrospective studies. We also used a standardized, time-limited room air challenge test (with oxygen saturation monitoring) to define BPD and reduce outcome detection bias. The single center aspect of the study meant that the same consensus-driven, standardized approaches to respiratory, hemodynamic, fluid, nutrition, and PDA evaluation and management were consistent among the infants.

In conclusion, we found no significant change in the incidence of BPD (grades 2 or 3) or death (or other secondary morbidities) when prophylactic indomethacin was used routinely in infants <25 weeks’ gestation.

## Data Availability

The datasets generated and/or analyzed during the current study are available from the corresponding author on reasonable request.
